# The Effect of Diagnostic Absorbed Doses from ^131^I on Human Thyrocytes *in Vitro*

**DOI:** 10.3390/ijms160714608

**Published:** 2015-06-29

**Authors:** Zbigniew Adamczewski, Mariusz Stasiołek, Bolesław Karwowski, Marek Dedecjus, Daria Orszulak-Michalak, Anna Merecz, Przemysław W. Śliwka, Bartosz Puła, Andrzej Lewiński

**Affiliations:** 1 Department of Endocrinology and Metabolic Diseases, Medical University of Lodz, 93-338 Lodz; Poland; E-Mail: zbadam@o2.pl; 2 Department of Endocrinology and Metabolic Diseases, Polish Mother’s Memorial Hospital—Research Institute, 93-338 Lodz, Poland; E-Mail: p.sliwka87@gmail.com; 3 Department of Neurology, Polish Mother’s Memorial Hospital—Research Institute, 93-338 Lodz, Poland; E-Mail: mstasiolek@yahoo.de; 4 Food Science Department, Medical University of Lodz, 90-151 Lodz, Poland; E-Mails: boleslaw.karwowski@umed.lodz.pl (B.K.); anna.merecz@umed.lodz.pl (A.M.); 5 Department of Oncological Endocrinology and Nuclear Medicine, Maria Skłodowska-Curie Memorial Cancer Center and Institute of Oncology, 02-781 Warsaw, Poland; E-Mail: marek.dedecjus@gmail.com; 6 Department of Biopharmacy, Medical University of Lodz, 90-151 Lodz, Poland; E-Mail: daria.orszulak-michalak@umed.lodz.pl; 7 Department of Hematology, Institute of Hematology and Transfusion Medicine, 02-776 Warsaw, Poland; E-Mail: bartosz.pula@gmail.com

**Keywords:** thyroid gland, ^131^I, thyroid stunning

## Abstract

Background: Administration of diagnostic activities of ^131^I, performed in order to detect thyroid remnants after surgery and/or thyroid cancer recurrence/metastases, may lead to reduction of iodine uptake. This phenomenon is called “thyroid stunning”. We estimated radiation absorbed dose-dependent changes in genetic material, in particular in sodium iodide symporter (*NIS*) gene promoter, and NIS protein level in human thyrocytes (HT). Materials and Methods: We used unmodified HT isolated from patients subjected to thyroidectomy exposed to ^131^I in culture. The different ^131^I activities applied were calculated to result in absorbed doses of 5, 10, and 20 Gy. Results: According to flow cytometry analysis and comet assay, ^131^I did not influence the HT viability in culture. Temporary increase of 8-oxo-dG concentration in HT directly after 24 h (*p* < 0.05) and increase in the number of AP-sites 72 h after termination of exposition to 20 Gy dose (*p* < 0.0001) were observed. The signs of dose-dependent DNA damage were not associated with essential changes in the NIS expression on mRNA and protein levels. Conclusions: Our observation constitutes a first attempt to evaluate the effect of the absorbed dose of ^131^I on HT. The results have not confirmed the theory that the “thyroid stunning” reduces the NIS protein synthesis.

## 1. Introduction

Thyroid cancer accounts for around 1% of all malignancies [1]. Diagnostic whole body scan (DxWBS) with radioiodine ^131^I, performed in order to detect thyroid tissue remaining after surgery and/or thyroid cancer metastases, belongs to the main elements of follow up examination in patients thyroidectomized due to the differentiated thyroid cancer (DTC) [2]. During DxWBS, ^131^I is administered orally at various diagnostic activities; most authors prefer a range of 74–185 MBq [3]. However, it is suggested that in some cases, administration of a diagnostic activity of radioiodine may lead to reduction of iodine uptake by remaining normal thyroid or DTC cells during the post-therapy whole body scan (RxWBS). This phenomenon is called “thyroid stunning”. It has been suggested that—as a result of this process—target cells (*i.e.*, DTC cells) may lose their responsiveness to subsequent radioiodine therapy. Due to this assumption, “thyroid stunning” is considered a significant clinical problem but informative studies on this subject are unclear in literature till now. Stunning was first described in 1951 [4] and further studies lead continuously to contradictory results on the subject in question. On one hand, not all experts have advocated that the stunning effect occurs [5,6,7], on the other, stunning has clearly been demonstrated by some authors [8,9,10,11,12]. It has been suggested that “thyroid stunning” may result from direct radiation damage of the cells exposed to the radiotracer, used previously for diagnostic purposes [13]. Additionally, it was demonstrated that irradiation of cells occurring as an effect of an uptake of diagnostic activities of ^131^I was associated with downregulation of the sodium-iodine symporter (NIS) expression, which—in consequence—diminished NIS function in living cells [14].

Although the authors of several reports have investigated “thyroid stunning” conditions *in vivo*, *i.e.*, in patients subjected to the radioiodine treatment [9,10,12,15,16,17,18,19,20,21,22,23], the molecular mechanisms of this phenomenon remain poorly characterized. The thyroid gland has the ability to concentrate iodide, which is then utilized to synthesize thyroglobulin and thyroid hormones. On the molecular level, this sequence of events is dependent on the activity of NIS, glycoprotein localized on apical membrane of thyroid follicular cells. Thyroid stimulating hormone (TSH) is believed to be the main factor regulating the accumulation of iodide in the thyroid and *NIS* gene expression in a mechanism involving the cAMP pathway [24,25]. Interestingly, the *in vitro* studies have shown the influence of standardized radiation absorption doses on the *NIS* gene expression in unchanged/native porcine thyroid cells, the other authors presented the reduction of iodine uptake by thyrocytes exposed to the absorbed doses in the range of 0.5–80 Gy [14,26,27,28]. Based on the molecular characteristics of thyroid function, we assume that the level of thyroid stunning may be associated with changes in NIS protein function or with the level of radioiodine dependent damages in the *NIS* gene structure [24].

Accordingly, the aim of the study was to characterize radiation dose-dependent changes in genetic material, in particular in *NIS* gene promoter and NIS protein level, of freshly isolated non-malignant human thyrocytes. The results—generated in models *in vitro*—based on immortalized thyrocyte-like cell lines—may be potentially strongly influenced by the “unnatural” molecular characteristics of these cells.

In the present study, we have designed the *ex vivo* experimental model as close as possible to the conditions expected on the level of local thyroid microenvironment in patients, subjected to the radioiodine procedures after surgery due to DTC. The basic assumption of the study was to conduct repetitive experiments on thyrocytes isolated in each single experiment from one individual subject in order to assess general phenomena and—at the same time—to get insight into interpersonal differences. We believe that the results obtained with this kind of the *ex vivo* model allow discussing the accuracy of currently used diagnostic-therapeutic algorithms in DTC.

## 2. Results

Multiple parameters were assessed in thyrocytes exposed to ^131^I in culture, in order to get insight into possible cellular and molecular mechanisms underlying the stunning phenomenon. The analysis encompassed measurements of extent apoptosis and necrosis of thyrocyte in culture, thyrocyte NIS expression on mRNA and protein level as well as selected DNA damage markers.

### 2.1. Apoptosis

**Figure 1:**
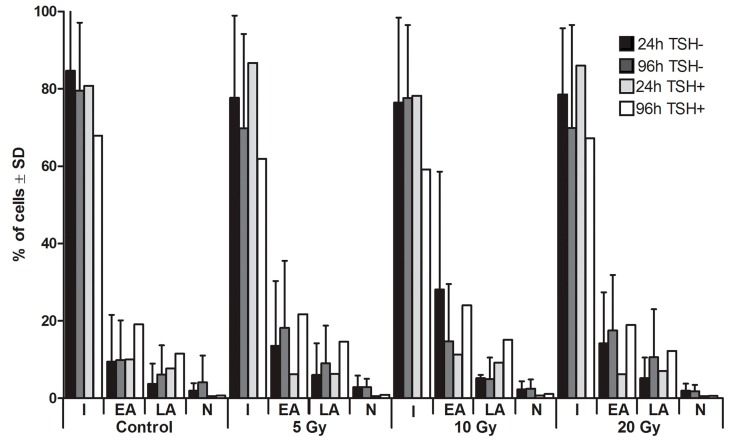
Survival of human thyrocytes in culture with ^131^I. The graph presents the percentages (±SD) of thyrocytes undergoing apoptotic or necrotic death processes, as assessed by flow cytometry directly after 24 h of ^131^I exposure (5, 10, 20 Gy) or after additional 72 h of culture without ^131^I. The culture was performed parallel with or without Thyroid stimulating hormone (TSH) stimulation. I—intact cells, EA—early apoptosis, LA—late apoptosis, N—necrosis.

**Figure 2:**
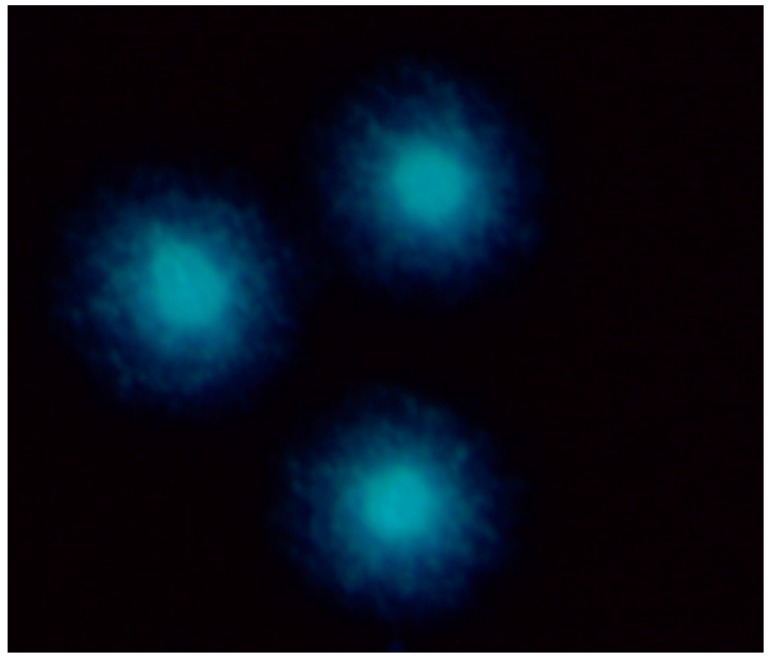
Representative images of DNA comets, obtained from human thyrocytes. The thyrocytes were stained with 4′,6-diamidino-2-phenylindole (DAPI), observed in fluorescent microscopy at magnification 400×. DNA damage was calculated as the DNA tail area/whole DNA area (%) and the comet tail length (from the center of DNA head to the end of the DNA tail). The picture shows intact cells without the DNA tail.

The percentage of thyrocytes in different stages of apoptotic and necrotic death process was assessed after 24 and 96 h of culture with flow cytometry on the basis of Annexin V and Propidium Iodide staining. Regardless of the culture conditions (^131^I absorbed dose; TSH presence) and duration, FACS analysis revealed repeatedly that more than 80% of cultured cells were intact. Moreover, we did not observe any influence of the applied absorbed doses of ^131^I on the rate of apoptosis and necrosis of thyrocytes *in vitro* (Figure 1). In order to confirm the results obtained with FACS analysis, in parallel experiments we also performed comet assay [29]. In all indicated time points and culture conditions, obtained images showed round, tight heads of DNA comets without a signs of fragmentation, characteristic for apoptotic process (Figure 2). Taken together, these results imply that administration of ^131^I, in the absorbed dose of 5 to 20 Gy did not influence the viability of thyrocytes in our experiments. Importantly, these observations allowed us to perform further analyses aimed at NIS expression and DNA damage markers without considering apoptosis rate as a possible result-influencing factor.

### 2.2. Expression of Sodium Iodide Symporter (NIS) Gene

**Figure 3:**
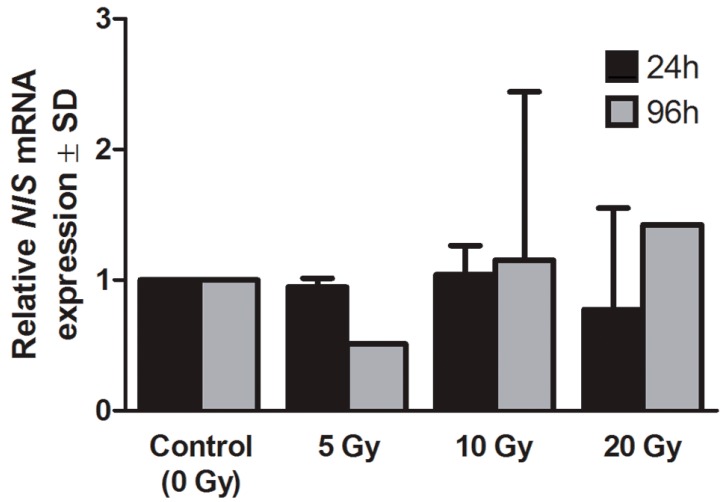
Expression of sodium iodide symporter (*NIS*) gene by human thyrocytes in culture with ^131^I. The graph shows relative *NIS* gene expression (±SD) obtained from human thyrocytes directly after 24 h of ^131^I exposure (5, 10, 20 Gy) or after additional 72 h of culture without ^131^I.

We used the RT-qPCR technique to measure the influence of beta and gamma radiation emitted by ^131^I on *NIS* gene expression. The thyroid cells did not show any statistically significant deregulation of *NIS* gene expression. The level of NIS mRNA in freshly isolated thyrocytes was found to be relatively low as compared to endogenous control (GAPDH) and remained stable after 24 h incubation with ^131^I. After 96 h of incubation, slight variations of *NIS* gene expression were observed, however those differences did not reach statistical significance (Figure 3).

### 2.3. Expression of NIS Protein

**Figure 4:**
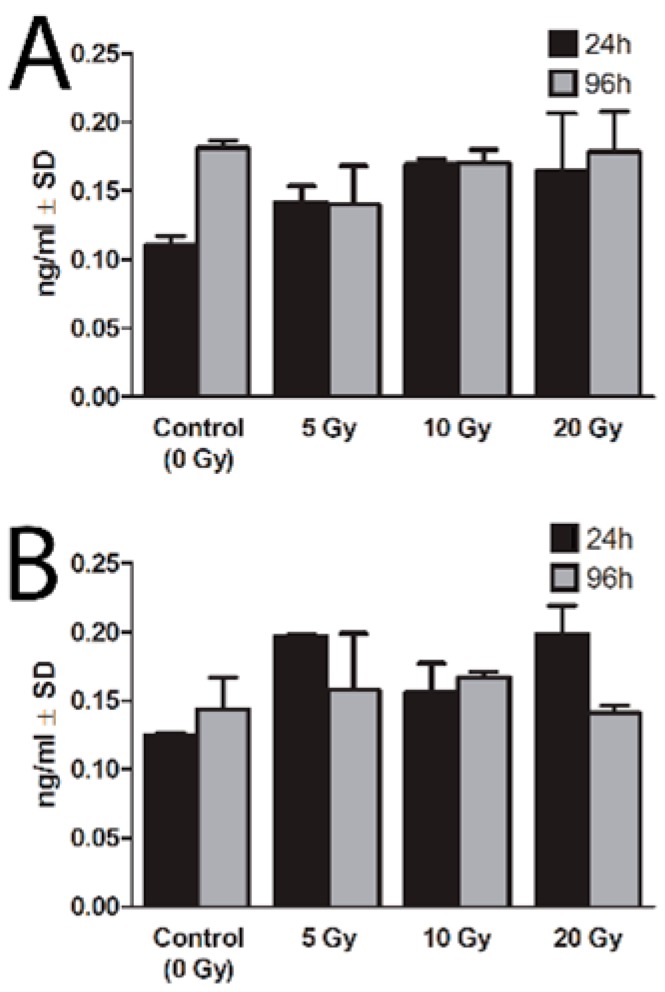
Expression of NIS protein in human thyrocytes cultured with ^131^I. The graph presents the mean NIS protein expression (ng/mL ± SD) in cultured thyrocytes directly after 24 h of ^131^I exposure (5, 10, 20 Gy) or after additional 72 h of culture without ^131^I. The culture was performed parallel with (**A**) or without (**B**) TSH stimulation.

The lack of radioiodine-associated changes in *NIS* gene expression does not exclude post-transcriptional mechanisms affecting the level of NIS protein in cultured thyrocytes protein. The NIS protein level was assessed using ELISA method in lysates obtained from thyrocytes exposed to radioiodine in culture. Similarly to *NIS* gene expression we did not find any significant changes in the presence of NIS protein in thyrocytes exposed for 24 h to various absorbed doses of ^131^I and cultured up to 96 h with or without TSH stimulation. The concentration of NIS protein in thyrocytes showed strong variations in particular time-points but we did not observe inhibitory effects of ^131^I exposition in any of the applied doses (Figure 4).

### 2.4. DNA Damage

#### 2.4.1. Comet Assay

As indicated earlier, the comet assay did not show any differences in the level of DNA damages between cells incubated with and without ^131^I. In all analyzed samples DNA content in comet tails did not exceed 1%, regardless of TSH stimulation and culture duration (Figure 2).

#### 2.4.2. 8-Oxo-7,8-dihydro-2′deoxyguanosine (8-oxo-dG)

**Figure 5:**
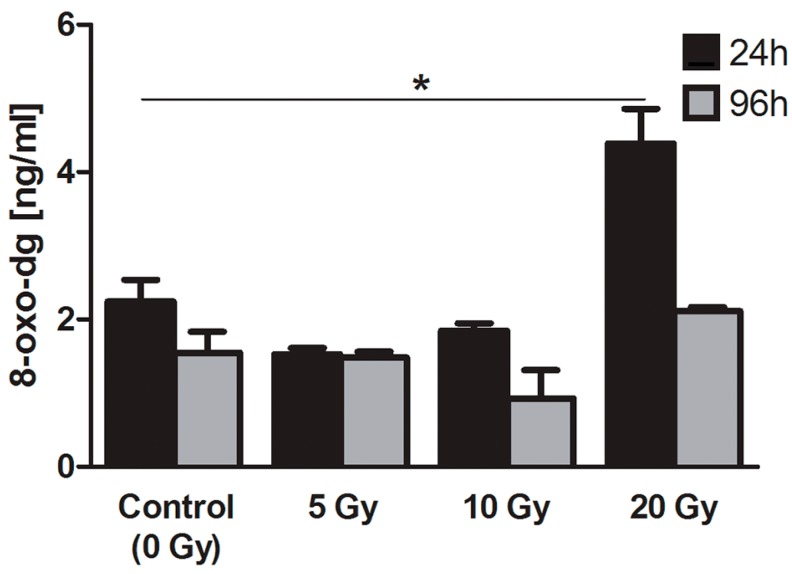
8-oxo-dG concentration in human thyrocytes cultured with ^131^I. The graph presents 8-oxo-dG concentration (ng/mL ± SD) in cultured thyrocytes directly after 24 h of ^131^I exposure (5, 10, 20 Gy) or after additional 72 h of culture without ^131^I (* *p* < 0.05).

8-oxo-dG is one of the most important DNA damage markers and can be considered as an indicator of cell condition in the case of radiation injury [30]. In our experiments, we found a significant two-fold increase of 8-oxo-dG concentration in thyrocytes directly after 24 h of being exposed to the highest absorbed dose of ^131^I (20 Gy) in culture. Importantly, after additional 72 h of culture without ongoing radiation injury, the 8-oxo-dG level returned to the values observed in control experiments. The lower absorbed doses (5 and 10 Gy) did not result in any significant changes in 8-oxo-dG concentration in cultured thyrocytes (Figure 5).

#### 2.4.3. Apurinic/Apirymidinic Sites (AP-Site)

**Figure 6:**
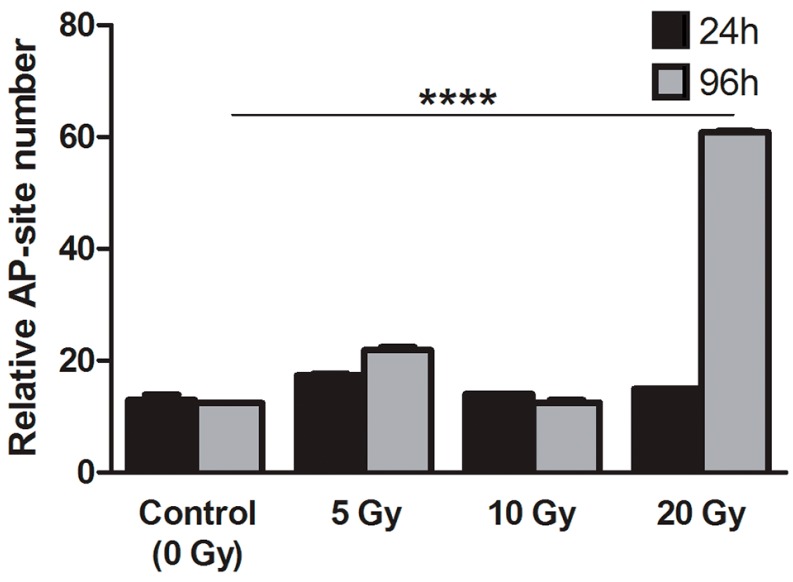
AP-site level in human thyrocytes cultured with ^131^I. The graph presents AP-site level (AP-sites/100,000 bp ± SD) in DNA measured in samples obtained from cultured thyrocytes directly after 24 h of ^131^I exposure (5, 10, and 20 Gy) or after additional 72 h of culture without ^131^I (**** *p* < 0.0001).

The number of AP-sites is considered to correlate with the intensity of DNA repair process. In our experiments, we found a significant five-fold increase in the number of AP-sites in cultured thyrocytes at 96 h time point (*p* < 0.0001), *i.e.*, 72 h after termination of exposition to highest absorbed dose of ^131^I (20 Gy) *in vitro*, as compared to control thyrocytes (Figure 6). Most interestingly, this difference was not observed directly after ^131^I exposure, suggesting an existence of DNA repair processes shifted in time in comparison to DNA injury indicated by increased 8-oxo-dG levels (see above).

## 3. Discussion

The literature data regarding the existence of “thyroid stunning” phenomenon are not unequivocal and frequently mutually excluding. Clinical observations indicating the presence of thyroid stunning are mostly based on the evaluation of the influence of ^131^I diagnostic dose (range 37–370 MBq), without the calculation of absorbed dose [10,12,16,17]. Park *et al.* [12] reported that stunning was present in 40% of investigated patients when the diagnostic activity of ^131^I was 111 MBq. However, since those authors reduced diagnostic activity to the 37–74 MBq, they have not observed obvious stunning effect in WBS [16]. Furthermore, in several studies the increased reduction in ^131^I uptake between DxWBS and RxWBS was not present when the diagnostic activity of ^131^I was equal to 185 MBq or less [6,31,32,33,34].

Our observations suggest that the patients who qualified to radioiodine remnant ablation, would absorb ^131^I doses ranging from 2.5 to 870 Gy after an administration of 111 MBq (diagnostic activity) [35]. Thus, in some cases the absorbed energy doses can be equal to the doses used in hyperthyroidism treatment or even could be recognized as ablative. This assumption seems to be supported by frequently observed thyroid remnants ablation in patients after DxWBS who were not qualified in order to ablate normal thyroid tissue after surgery because of DTC. Taking into consideration the above mentioned observations, we can suspect that conclusions regarding the effect of ^131^I diagnostic activity on the appearance of “thyroid stunning”, drawn without the knowledge of the absorbed dose, are strongly affected by interpersonal dosimetry differences and do not reflect the real association of “thyroid stunning” phenomenon with the activity of ^131^I.

Accordingly, Jeevanran and colleagues [9] demonstrated that subsequent thyroid uptake of iodine was—by average—reduced by 75% after an initial diagnostic activity, in which the dose absorbed by thyroid remnants was 35 Gy or more. On the other hand, the use of absorbed dose of 17.5 Gy for diagnostic purposes reduced radioiodine uptake by 25% only.

It should be emphasized that even the strict characterization of dosimetric parameters enables the assessment of radiosensitivity of thyroid remnants with some approximation only [36]. In many cases of DTC, thyroid cancer represents only one out of many different pathologies of the thyroid gland. Thus, functional properties of thyroid remnants to great extent depend on the nature of co-existing pathology. Observations from radioiodine treatment of hyperthyroidism cases indicate that radiosensitivity of thyrocytes depended strongly on the nature of underlying thyroid disease [37]. Most probably, similar relationship may be expected also in postoperative thyroid remnants harboring different kinds of cellular pathology, which would explain substantial proportion of ablations in patients with DTC after surgery who received low ^131^I activities (1110 MBq). Moreover, the variability of reported findings, including differences in quantitative analysis of radioiodine uptake, as well as qualitative radioiodine thyroid uptake assessment by DxWBS in comparison to RxWBS, can result from unstandardized protocols used in clinical nuclear medicine.

Therefore, the reported decrease of radioiodine uptake can be in some cases attributed to the death of significant part of thyroid cells, which can lead to “stunning effect” imitation, without any influence on the future effect of radioiodine therapy. Alternatively, radioiodine may induce a destructive thyroiditis, which can cause iodine release from thyroid remnants. In this situation, the concentration of iodine in blood serum increases, whereas the uptake of radioiodine decreases as the competitive effect.

Our study was designed to reflect, as much as possible, the situation known in the clinical practice. In order to achieve this purpose, we used in our experiments unmodified human thyrocytes exposed to ^131^I in culture. The radiation doses applied in our study were similar to doses absorbed in most cases by thyroid remnants during DxWBS. In these experimental settings, we demonstrated that ^131^I (5–20 Gy) did not influence the viability of human thyrocytes in culture.

In further analysis, we showed that the ^131^I-associated irradiation led to the significant increase of 8-oxo-dG concentration in thyrocytes directly after 24 h exposition to the highest absorbed dose (20 Gy). The 8-oxo-dG level returned to the values observed in control experiments following 72 h of culture without radiation injury, which suggests a temporary nature of the DNA damage. This assumption seems to be supported by the results of AP-sites analysis. Significant increase of the number of AP-sites was found in thyrocytes 72 h after termination of exposition to highest absorbed dose of ^131^I (20 Gy) *in vitro*. This result is agreeable with results obtained for 8-oxo-dG after 24 h. The time-shift in the increase of these two DNA damage markers (8-oxo-dG and AP-site; 24 and 96 h, respectively) can be explained by the mechanism of 8-oxo-dG repair, *i.e.*, base excision repair (BER). AP-sites are considered as transition products of those processes and thus considered as DNA-repair markers [38,39]. It should also be noted that these repair mechanisms are not specific for 8-OHdG, and the increase of AP-sites could result from multiple, possibly separated in time, mechanisms of DNA-damage caused by the ionizing radiation and/or free radicals. Importantly, it cannot be excluded that the DNA repair processes occur with a significant delay in irradiated thyrocytes [40] (Figure 6).

In the light of the “thyrocyte stunning” theory, it is of great importance that the signs of absorbed dose dependent DNA damage in our experiments were not associated with significant changes in the expression of NIS on the mRNA and protein level. Interestingly, the concentration of NIS mRNA and NIS protein in thyrocytes varied significantly between particular donors and there was no specific pattern of changes of that parameter in response to ^131^I exposition.

Significant differences observed in thyroid cells derived from different patients, indicate that the obtained results depend on individual cells radiosensitivity. This may cause that the outcomes of each experiment conducted on human thyrocytes, will be dependent on the individual features of the cells, *i.e.*, the outcomes will be the consequence of random patients selection.

In this context, our study, being—according to our knowledge—the first published report in which effect of the absorbed dose of ^131^I on human thyrocytes *in vitro* has been evaluated, brought the results, demonstrating that the transfer of the idea of “thyroid stunning” into clinical practice could be very difficult. In conclusion, this study performed on human thyrocytes did not confirm reduction of NIS protein synthesis as a mechanism underlying “thyroid stunning” phenomenon.

## 4. Experimental Section

### 4.1. Ethics Statement

This study has been approved by Polish Mother’s Memorial Hospital—Research Institute Ethical Committee in Lodz, Poland (No. 88/2014, December 2014). All the study participants gave informed written consent for the surgery procedure and for laboratory examination of the postoperative material samples. In all cases, full histopathological examination of thyroid surgery specimens was performed according to good clinical practice and medical standards. After the histopathological examination, the rest of the biological material was used for the thyrocyte isolation.

### 4.2. Thyrocyte Isolation and Culture

Thyroid tissue samples were obtained from patients subjected to thyroidectomy because of standard indications including: non-toxic nodular goiter, toxic nodular goiter, Graves’ disease and Hashimoto’s disease (*n* = 17). Thyrocytes were isolated from tissue fragments unaffected by macroscopic pathological processes. The thyroid tissue was sliced and minced through 100 µm pore cell strainer (BD Falcon, Franklin Lakes, NJ, USA) and washed in HBSS (Life Technologies, Carlsbad, CA, USA). Cells were seeded in 24-well plates at density of 1 × 10^7^ cell per well and were cultured in humidified atmosphere (5% CO_2_) at 37 °C in Heraeus Type UT6 incubator (Kendro Laboratory Products, Hanau, Germany). The culture medium contained RPMI 1640 with l-glutamine (Life Technologies) enriched with 5% fetal calf serum (Sigma-Aldrich, Saint Louis, MO, USA) and supplemented with: Gibco^®^ Antibiotic-Antimycotic solution (contains 10,000 units/mL of penicillin, 10,000 µg/mL of streptomycin, and 25 µg/mL of Fungizone^®^ (Life Technologies)), kanamycin (100 µg/mL), transferrin (6 μg/mL), human recombinant insulin (10 μg/mL), somatostatin (10 ng/mL) and Gly-His-Lys (10 ng/mL) (all from Sigma-Aldrich, Saint Louis, MO, USA). The thyrocytes culture was performed in the presence of ^131^I applied in various doses encompassing values expected in diagnostic applications (range of absorbed energy dose: 5–20 Gy). The influence of thyrotropin (thyroid stimulating hormone, TSH) on analyzed parameters was evaluated in cultures supplemented with bovine pituitary TSH (Sigma-Aldrich, Saint Louis, MO, USA) (30 µU/mL). Due to the relatively short time of culture and culture methodology (cells were resuspended), we did not observe any differences in cytoarchitectonics between thyrocytes cultured in the absence and presence of TSH. After 24 h, cells were harvested and divided for the direct analysis or further culture in fresh medium without ^131^I (cumulative culture time 96 h). In parallel control experiments, thyrocytes underwent exactly the same culture procedures without ^131^I.

### 4.3. Fluorescence-Activated Cell Sorting (FACS) Analysis

Flow cytometry apoptosis analysis was performed with Annexin V Apoptosis Detection Kit FITC Enzyme (eBioscience, San Diego, CA, USA) assay using Annexin V and Propidium iodide (PI) according to manufacturer’s protocol. 1 × 10^5^ cells in each sample were used for staining. Analysis was performed with FACSCanto II cytometer using FACSDiva software (BD Bioscience, Franklin Lakes, NJ, USA). At least 2 × 10^4^ cells were counted and the percentage of cells in the state of early apoptosis (EA) (recognized as PI^low^, Annexin V^low^), late apoptosis (LA) (PI^high^, Annexin V^high^) or necrosis (N) (PI^−^, Annexin V^high^) was measured.

### 4.4. RT-qPCR

Total RNA was extracted from cultured cells at the indicated time points with the QIAGEN RNeasy Mini Kit (Qiagen, Venlo, The Netherlands), according to the manufacturer’s protocol. UV spectrophotometry was used for quantification of isolated RNA. For each sample, cDNA was synthetized using random hexamers and QuantiTect Reverse Transcription Kit (Qiagen). The relative quantitative PCR analysis was performed with QuantiTect SYBR^®^ Green PCR Kit (Qiagen) and the Applied Biosystems^®^ 7500 Real-Time PCR System (Life Technologies). Oligonucleotide primers for the human *NIS* gene and for reference gene (*GAPDH*) were purchased from Metabion (Martinsried, Germany). Primer sequences were as follows: NIS 5′-TCTCTCAGTCAACGCCTCT-3′ (forward) and 5′-ATCCAGGATGGCCACTTCTT-3′ (reverse); GAPDH 5′-CACCTTCCCCATGGTGTCT-3′ (forward) and 5′-CCCCGGTTTCTATAAATTGAGC-3′ (reverse). All samples were done in triplets.

### 4.5. Comet Assay

The comet assay was carried out under alkaline conditions according to Singh *et al.* [29], with the following modifications. Cells were suspended in Low Melting Point (LMP) agarose (0.75% in PBS) and spread on microscope slides precoated with 0.5% normal agarose. Slides were then put in lysis solution (2.5 M NaCl, 0.1 M EDTA, 10 mM Tris and 1% Triton X-100, pH 10) for 1 h at 4 °C, and then incubated in an electrophoresis buffer (0.3 M NaOH and 1 mM EDTA, pH 13) for 20 min to allow unwinding of DNA. Electrophoresis was carried out for 20 min at 0.7 V/cm (30 mA). After electrophoresis, slides were washed in neutralization buffer (0.4 M Tris, pH 7.5), dried, stained with 2 µg/mL of a fluorescent stain—4′,6-diamidino-2-phenylindole (DAPI), and covered with a coverslip.

Preparations were viewed and analyzed under 400× magnification. Images of comets for analysis were obtained using a JENOPTIK camera (Jenoptik, Jena, Germany), equipped with UV filter block (excitation filter (359 nm) and barrier filter (461 nm)) connected to a fluorescent microscope (Delta Optical, Minsk Mazowiecki, Poland). Slides were scored using image analysis system, CaspLab v. 1.2.3β1 (University of Wroclaw, Institute of Theoretical Physics, Wroclaw, Poland) [41]. Measurements were made for 50 cells per analyzed slide after image archiving. The comet tail formation was analyzed as a quantitative measure of the DNA damage and the differences between cells were estimated on the basis of that parameter (percentages of damage in cell tail—control *versus* irradiated one). All the values in this study were expressed as mean ± standard error of mean (SEM). Differences between mean values were tested for using the One-Way ANOVA test.

### 4.6. 8-Oxo-7,8-dihydro-2'deoxyguanosine (8-oxo-dG) Measurement

Levels of 8-oxo-dG, a DNA damage biomarker, were determined in total DNA isolated from cultured thyrocytes using OxiSelect™ Oxidative DNA Damage ELISA Kit (8-OHdG Quantitation) (Cell Biolabs, San Diego, CA, USA) according to manufacturer’s protocol. Total DNA was isolated from cells with DNAzol (Life Technologies). UV spectrophotometry was used for quantification of isolated DNA. The extracted DNA was then denaturated and cut with nuclease P1 (Sigma-Aldrich, Saint Louis, MO, USA) followed by incubation with alkaline phosphatase. The samples were then centrifuged (6000 rds./min, 5 min) and supernatants were used for further analysis. Fifty microliters of sample or standards (0 to 20 ng/mL of 8-oxo-dG) was applied on 96-well plate, incubated 10 min at room temperature and then 50 µL of anti-8-oxo-dG antibody was added. After an enzymatic reaction, detection was performed in Multilabel Plate Reader Victor X (Perkin Elmer, MA, USA). Optical density at 450 nm was measured and 8-oxo-dG concentration in each sample was calculated according to standards.

### 4.7. Apurinic/Apirymidinic Sites (AP-Site) Determination

AP-sites were determined in total DNA isolated from cultured thyrocytes using OxiSelect™ Oxidative DNA Damage Quantitation Kit (Cell Biolabs) according to manufacturer’s protocol. Total DNA was isolated as described previously DNA samples were incubated with aldehyde reactive probe (ARP) solution for 1 h at 37 °C. Next, 90 µL of TE buffer, 1 µL glycogen, 10 µL sodium acetate and 300 µL of 98% ethanol were added followed by 30 min incubation at −20 °C. After centrifugation, supernatants were removed and sediments were reconstituted in TE buffer. Fifty microliters of DNA solution in TE buffer (1 µg/mL) from each sample or standard DNA solution (0 to 40 AP-sites/100,000 base pairs) were applied on 96-well plate, supplemented with 50 µL of binding buffer and incubated overnight at room temperature. After an enzymatic reaction, detection was performed in Multilabel Plate Reader Victor X (Perkin Elmer, Waltham, MA, USA). Optical density at 450 nm was measured and the number of AP-sites in each sample was calculated according to standards.

### 4.8. Enzyme-Linked Immunoabsorbent Assay (ELISA)

NIS protein level was measured in lysates from cultured thyreocytes using Enzyme-Linked Immunoabsorbent Assay Kit for Sodium-Iodide Symporter (USCN Life Science, Wuhan, China) according to manufacturer’s protocol. Harvested thyrocytes were suspended in PBS at density 1.5 × 10^6^ cells/mL and frozen at −20 °C and thawed 3 times. Samples were then centrifuged at 1400 rds./min for 15 min. Supernatants were used in further analysis. One hundred microliters of each supernatant and standards (0 to 200 ng/mL) were placed on 96-well plate and incubated for 2 h at 37 °C. Supernatants were next removed and 100 µL of reagent A for 1 h in 37 °C was applied. After washing procedure 100 µL of reagent B was applied for 30 min at 37 °C. After additional washing, 90 µL of substrate solution was added and samples were incubated 20 min at room temperature next, 50 µL of stopping solution was added and samples were analyzed in Multilabel Plate Reader Victor X (Perkin Elmer, Waltham, MA, USA). Optical density at 450 nm was measured and NIS protein concentration in each sample was calculated according to standards.

### 4.9. Statistical Analysis

Statistical analysis was performed using Prism 5.0 (GraphPad, La Jolla, CA, USA). In all the analyses a two-tailed unpaired *t*-test was utilized and the results are presented as mean ± standard deviation (SD). Differences were considered significant for *p* < 0.05 in all the analyses.

## References

[1] Pellegriti G., Frasca F., Regalbuto C., Squatrito S., Vigneri R. (2013). Worldwide increasing incidence of thyroid cancer: Update on epidemiology and risk factors. J. Cancer Epidemiol..

[2] Durante C., Costante G., Filetti S. (2013). Differentiated thyroid carcinoma: Defining new paradigms for postoperative management. Endocr. Relat. Cancer.

[3] Pacini F., Schlumberger M., Dralle H., Elisei R., Smit J.W., Wiersinga W., the European Thyroid Cancer Taskforce (2006). European consensus for the management of patients with differentiated thyroid carcinoma of the follicular epithelium. Eur. J. Endocrinol..

[4] Rawson R.W., Rall J.E., Peacock W. (1951). Limitations and indications in the treatment of cancer of the thyroid with radioactive iodine. J. Clin. Endocrinol. Metab..

[5] McDougall I.R. (1997). 74 MBq radioiodine ^131^I does not prevent uptake of therapeutic doses of ^131^I (*i.e.*, it does not cause stunning) in differentiated thyroid cancer. Nucl. Med. Commun..

[6] Morris L.F., Waxman A.D., Braunstein G.D. (2001). The nonimpact of thyroid stunning: Remnant ablation rates in ^131^I-scanned and nonscanned individuals. J. Clin. Endocrinol. Metab..

[7] Silberstein E.B. (2007). Comparison of outcomes after ^123^I *versus*
^131^I preablation imaging before radioiodine ablation in differentiated thyroid carcinoma. J. Nucl. Med..

[8] Lassmann M., Luster M., Hanscheid H., Reiners C. (2004). Impact of ^131^I diagnostic activities on the biokinetics of thyroid remnants. J. Nucl. Med..

[9] Jeevanram R.K., Shah D.H., Sharma S.M., Ganatra R.D. (1986). Influence of initial large dose on subsequent uptake of therapeutic radioiodine in thyroid cancer patients. Int. J. Radiat. Appl. Instrum. B.

[10] Muratet J.P., Daver A., Minier J.F., Larra F. (1998). Influence of scanning doses of iodine-131 on subsequent first ablative treatment outcome in patients operated on for differentiated thyroid carcinoma. J. Nucl. Med..

[11] Lees W., Mansberg R., Roberts J., Towson J., Chua E., Turtle J. (2002). The clinical effects of thyroid stunning after diagnostic whole-body scanning with 185 MBq ^131^I. Eur. J. Nucl. Med. Mol. Imaging.

[12] Park H.M., Perkins O.W., Edmondson J.W., Schnute R.B., Manatunga A. (1994). Influence of diagnostic radioiodines on the uptake of ablative dose of iodine-131. Thyroid.

[13] Medvedec M. (2005). Thyroid stunning *in vivo* and *in vitro*. Nucl. Med. Commun..

[14] Lundh C., Norden M.M., Nilsson M., Forssell-Aronsson E. (2007). Reduced iodide transport (stunning) and DNA synthesis in thyrocytes exposed to low absorbed doses from ^131^I *in vitro*. J. Nucl. Med..

[15] Park H.M. (1992). Stunned thyroid after high-dose I-131 imaging. Clin. Nucl. Med..

[16] Park H.M., Park Y.H., Zhou X.H. (1997). Detection of thyroid remnant/metastasis without stunning: An ongoing dilemma. Thyroidology.

[17] Huic D., Medvedec M., Dodig D., Popović S., Ivancević D., Pavlinovic Z., Zuvic M. (1996). Radioiodine uptake in thyroid cancer patients after diagnostic application of low-dose ^131^I. Nucl. Med. Commun..

[18] Yeung H.W., Humm J.L., Larson S.M. (2000). Radioiodine uptake in thyroid remnants during therapy after tracer dosimetry. J. Nucl. Med..

[19] Leger F.A., Izembart M., Dagousset F., Barritault L., Baillet G., Chevalier A., Clerc J. (1998). Decreased uptake of therapeutic doses of iodine-131 after 185-MBq iodine-131 diagnostic imaging for thyroid remnants in differentiated thyroid carcinoma. Eur. J. Nucl. Med..

[20] Verburg F.A., Verkooijen R.B., Stokkel M.P., van Isselt J.W. (2009). The success of ^131^I ablation in thyroid cancer patients is significantly reduced after a diagnostic activity of 40 MBq ^131^I. Nuklearmedizin.

[21] Wu H.S., Hseu H.H., Lin W.Y., Wang S.J., Liu Y.C. (2005). Decreased uptake after fractionated ablative doses of iodine-131. Eur. J. Nucl. Med. Mol. Imaging.

[22] Arad E., Flannery K., Wilson G.A., O’Mara R.E. (1990). Fractionated doses of radioiodine for ablation of postsurgical thyroid tissue remnants. Clin. Nucl. Med..

[23] Leger A.F., Pellan M., Dagousset F., Chevalier A., Keller I., Clerc J. (2005). A case of stunning of lung and bone metastases of papillary thyroid cancer after a therapeutic dose (3.7 GBq) of ^131^I and review of the literature: Implications for sequential treatments. Br. J. Radiol..

[24] Dohán O., de la Vieja A., Paroder V., Riedel C., Artani M., Reed M., Ginter C.S., Carrasco N. (2003). The sodium/iodide symporter (NIS): Characterization, regulation, and medical significance. Endocr. Rev..

[25] Kogai T., Brent G.A. (2012). The sodium iodide symporter (NIS): Regulation and approaches to targeting for cancer therapeutics. Pharmacol. Ther..

[26] Nordén M.M., Larsson F., Tedelind S., Carlsson T., Lundh C., Forssell-Aronsson E., Nilsson M. (2007). Down-regulation of the sodium/iodide symporter explains ^131^I-induced thyroid stunning. Cancer Res..

[27] Postgard P., Himmelman J., Lindencrona U., Bhogal N., Wiberg D., Berg G., Jansson S., Nyström E., Forssell-Aronsson E., Nilsson M. (2002). Stunning of iodide transport by ^131^I irradiation in cultured thyroid epithelial cells. J. Nucl. Med..

[28] Lundh C., Lindencrona U., Postgård P., Carlsson T., Nilsson M., Forssell-Aronsson E. (2009). Radiation-induced thyroid stunning: differential effects of ^123^I, ^131^I, ^99m^Tc, and ^211^At on iodide transport and NIS mRNA expression in cultured thyroid cells. J. Nucl. Med..

[29] Singh N.P., McCoy M.T., Tice R.R., Schneider E.L. (1988). A simple technique for quantitation of low levels of DNA damage in individual cells. Exp. Cell Res..

[30] Cadet J., Loft S., Olinski R., Evans M.D., Bialkowski K., Richard Wagner J., Dedon P.C., Møller P., Greenberg M.M., Cooke M.S. (2012). Biologically relevant oxidants and terminology, classification and nomenclature of oxidatively generated damage to nucleobases and 2-deoxyribose in nucleic acids. Free Radic. Res..

[31] Cholewinski S.P., Yoo K.S., Klieger P.S., O’Mara R.E. (2000). Absence of thyroid stunning after diagnostic whole-body scanning with 185 MBq ^131^I. J. Nucl. Med..

[32] Bajén M.T., Mañé S., Muñoz A., García J.R. (2000). Effect of a diagnostic dose of 185 MBq ^131^I on postsurgical thyroid remnants. J. Nucl. Med..

[33] Rosário P.W., Barroso A.L., Rezende L.L., Padrão E.L., Maia F.F., Fagundes T.A., Purisch S. (2005). 5 mCi pretreatment scanning does not cause stunning when the ablative dose is administered within 72 h. Arq. Bras. Endocrinol. Metab..

[34] Karam M., Gianoukakis A., Feustel P.J., Cheema A., Postal E.S., Cooper J.A. (2003). Influence of diagnostic and therapeutic doses on thyroid remnant ablation rates. Nucl. Med. Commun..

[35] Adamczewski Z., Makarewicz J., Lewinski A. (2007). Effects of absorbed dose of ^131^I isotope on the effectiveness of ablation of thyroid remnant tissue. Arch. Med. Sci..

[36] McCready V.R. (1995). A different approach to the use of unsealed radionuclides for cancer therapy. Nucl. Med..

[37] Riccabona G. (1994). ^131^I Therapy of Thyroid Disease: Why? When? How?.

[38] Dahlmann H.A., Vaidyanathan V.G., Sturla S.J. (2009). Investigating the biochemical impact of DNA damage with structure-based probes: Abasic sites, photodimers, alkylation adducts, and oxidative lesions. Biochemistry.

[39] Slupphaug G., Kavli B., Krokan H.E. (2003). The interacting pathways for prevention and repair of oxidative DNA damage. Mutat. Res..

[40] Maynard S., Schurman S.H., Harboe C., de Souza-Pinto N.C., Bohr V.A. (2009). Base excision repair of oxidative DNA damage and association with cancer and aging. Carcinogenesis.

[41] Dikilitas M., Kocyigit A. (2010). Mononuclear leukocyte DNA damage on higher cells caused by eco-friendly pesticides and their analysis using CAPS^®^ programme. J. Agric. Fac..

